# Supplementary figures and images for: A pro-oxidant combination of resveratrol and copper down-regulates multiple biological hallmarks of ageing and neurodegeneration in mice
Source: Sci Rep. 2022 Oct 14;12:17209. doi: 10.1038/s41598-022-21388-w (PMC9568542; doi:10.1038/s41598-022-21388-w)

## Supplementary Figure S1

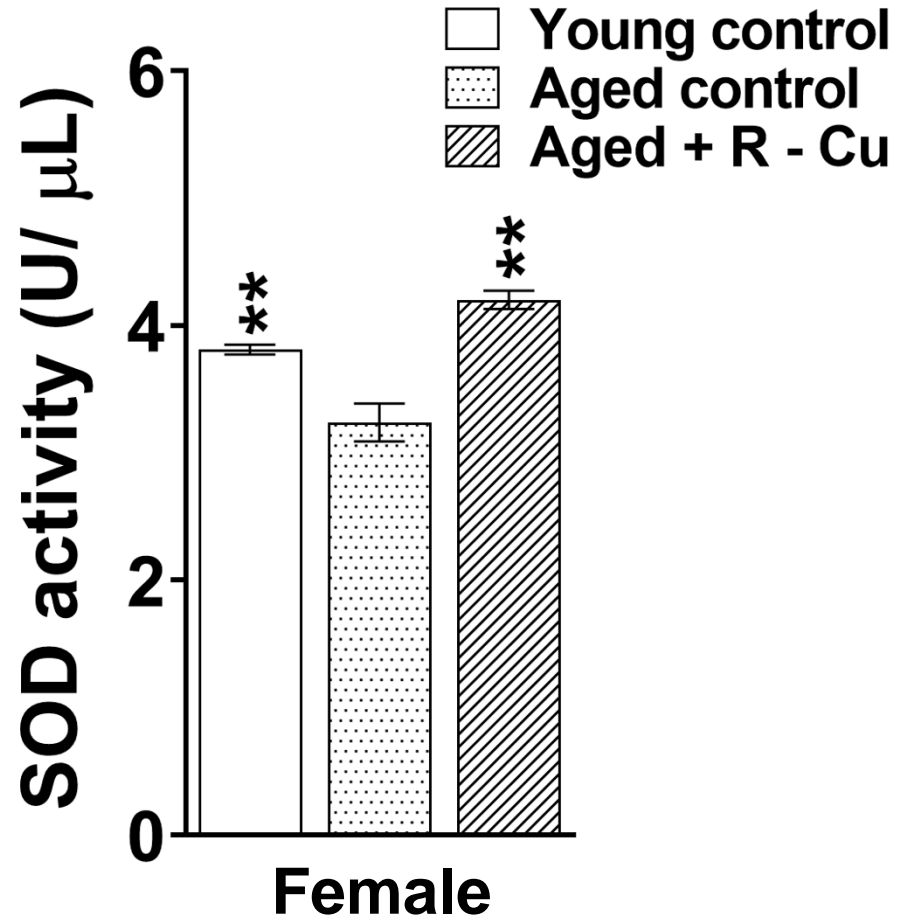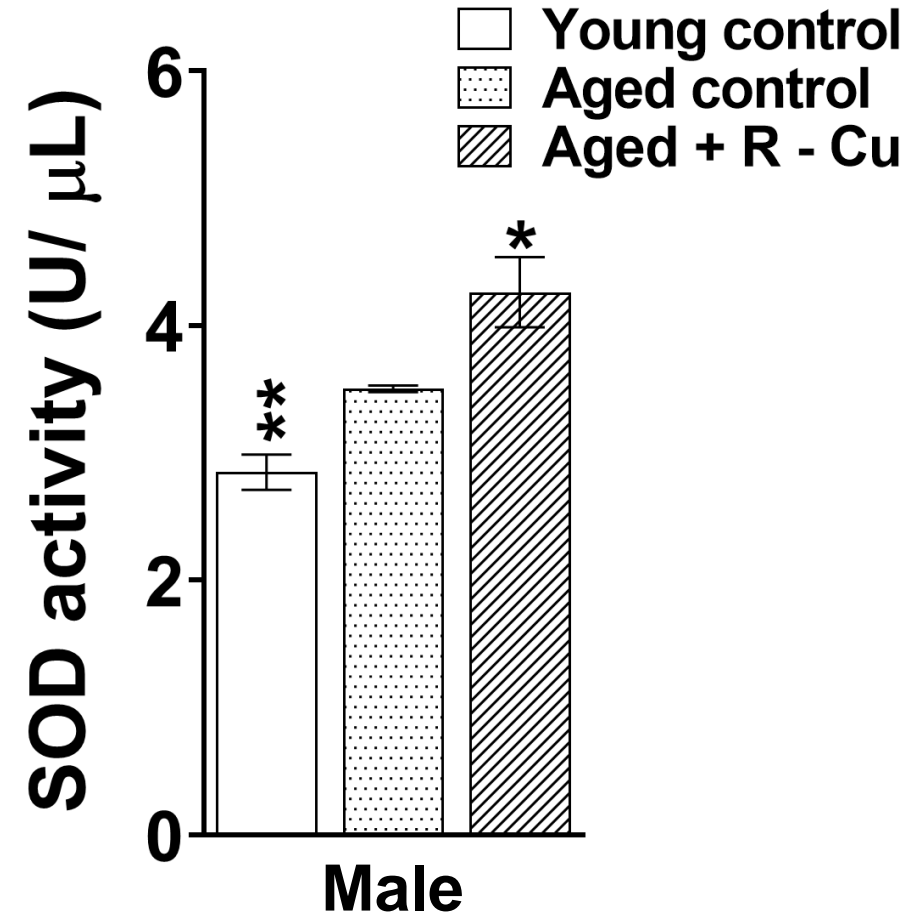

Supplement: Supplementary file 1 — Supplementary Figure S1. [file 41598_2022_21388_MOESM1_ESM.pdf]
