# Supplementary material for: A pro-oxidant combination of resveratrol and copper down-regulates multiple biological hallmarks of ageing and neurodegeneration in mice
Source: Sci Rep. 2022 Oct 14;12:17209. doi: 10.1038/s41598-022-21388-w (PMC9568542; doi:10.1038/s41598-022-21388-w)
Supplement: Supplementary file 2 — Supplementary Figure Legend. [file 41598_2022_21388_MOESM2_ESM.docx]

**Legend to Supplementary Figure S1**

R-Cu treatment upregulates SOD activity in serum. Bars represent mean ± SEM values of the four animals in each group of either sex. Statistical analysis was performed by two-tailed Student’s t test. * p < 0.05; ** p < 0.01, *** p < 0.001.
