# Supplementary material for: A pro-oxidant combination of resveratrol and copper down-regulates multiple biological hallmarks of ageing and neurodegeneration in mice
Source: Sci Rep. 2022 Oct 14;12:17209. doi: 10.1038/s41598-022-21388-w (PMC9568542; doi:10.1038/s41598-022-21388-w)
Supplement: Supplementary file 3 — Supplementary Table 1. [file 41598_2022_21388_MOESM3_ESM.docx]

**Supplementary Table 1**

**Description of Kits**

| **Sr. No.** | **Kits** | **Catalogue No.** | **Company / Vendor** |
| --- | --- | --- | --- |
| 1. | QIAamp DNA FFPE Tissue Kit (50) | 56404 | Qiagen, Hilden, Germany |
| 2. | Total BDNF Quantikine ELISA Kit | DBNT00 | R&D Systems, MN, USA |
| 3. | Mouse C-Reactive Protein /CRP Quantikine ELISA Kit | MCRP00 | R&D Systems, MN, USA |

**Description of Antibodies**

| **Sr. No.** | **Antibody / Probes** | **Catalogue No.** | **Company / Vendor** |
| --- | --- | --- | --- |
| 1. | Histone H4 IgG | Custom synthesized | Bioklone Biotech Pvt Ltd, Chennai, India |
| 2. | Anti-DNA Antibody | NB110- 89473 | Novus Biologicals, Colorado, USA |
| 3. | Anti- γ H2AX (Phospho S139) Antibody | ab26350 | Abcam, Cambridge, UK |
| 4. | Anti NF-kB p65 Antibody | ab32536 | Abcam, Cambridge, UK |
| 5. | Anti-53BP1 Antibody (EPR2172(2)) | ab175933 | Abcam, Cambridge, UK |
| 6. | Anti-CDKN2A/p16INK4a Antibody (2D9A12) | ab54210 | Abcam, Cambridge, UK |
| 7. | Anti-Cleaved Caspase-3 Antibody | ab2302 | Abcam, Cambridge, UK |
| 8. | Anti-TOMM20 Antibody (EPR15581-54) | ab186735 | Abcam, Cambridge, UK |
| 9. | Purified Anti-β-Amyloid, 1-42 Antibody | 805501 | BioLegend, California, USA |
| 10. | Anti-Superoxide Dismutase 1 Antibody | ab13498 | Abcam, Cambridge, UK |
| 11. | Anti-PML Antibody | P6746 | Merck-Millipore Sigma, Germany |
| 12. | Goat Anti-Rabbit IgG (H+L) FITC Conjugate Secondary Antibody | AP307F | Merck-Millipore Sigma, Germany |
| 13. | Goat Anti-Mouse IgG H&L (FITC) Pre-Adsorbed Secondary Antibody | ab7064 | Abcam, Cambridge, UK |
| 14. | Rabbit Anti-Goat IgG H&L (FITC) Secondary Antibody | ab6737 | Abcam, Cambridge, UK |
| 15. | Goat Anti-Mouse IgG H& L (FITC) Secondary Antibody | ab6785 | Abcam, Cambridge, UK |
| 16. | PNA Telomere FISH Probe ( Tel-G-Cy3) | F1006 | Panagene, South Korea |
| 17. | Single Chromosome Paint Probes Chromosome 7 Red (Ready to use) | Custom synthesized | Applied Spectral Imaging, Israel |
| 18. | Single Chromosome Paint Probes Chromosome 16 Green (Ready to use) | Custom synthesized | Applied Spectral Imaging, Israel |
