# Supplementary material for: A pro-oxidant combination of resveratrol and copper down-regulates multiple biological hallmarks of ageing and neurodegeneration in mice
Source: Sci Rep. 2022 Oct 14;12:17209. doi: 10.1038/s41598-022-21388-w (PMC9568542; doi:10.1038/s41598-022-21388-w)
Supplement: Supplementary file 4 — Supplementary Table 2. [file 41598_2022_21388_MOESM4_ESM.docx]

**Supplementary table 2**

**The ARRIVE Guidelines Checklist**

**Animal Research: Reporting In Vivo Experiments**

|  | **Item** | **Recommendation** | **Paragraph / Section** |
| --- | --- | --- | --- |
| **Title** |  | A pro-oxidant combination of resveratrol and copper down-regulates multiple biological hallmarks of ageing and neurodegeneration | **Title Page**  Page no 1 |
| **Abstract** |  | **Background**  Billions of cells die in the body every day, and cell-free chromatin particles (cfChPs) which are released from them enter into the extracellular compartments of the body, including into the circulation. We have reported that cfChPs can readily enter into healthy cells to damage their DNA, and activate apoptotic pathways and inflammatory cytokines. We hypothesized that repeated lifelong assault on healthy cells by cfChPs is the underlying cause of ageing, and that ageing can be retarded by deactivating cfChPs. The latter can be effected by oxygen radicals that are generated upon admixing the nutraceuticals resveratrol (R) and copper (Cu).  **Methods**  The study comprised of 24 C57Bl/6 mice (12 male, 12 female). Four mice of either sex acted as young controls and were sacrificed when they were 3 months old; their brain and blood were collected. The remaining 16 mice (8 male and 8 female) were allowed to age until they were 10 months old and divided into: 1) Ageing control mice (N=4 of each sex), and 2) R-Cu treated ageing mice (N=4 of each sex). Animals of both groups were sacrificed after 12 months, at age 22 months; their brain and blood were collected. R-Cu was administered twice daily by oral gavage for 12 months (from 10 months to 22 months) at a dose of 1mg/Kg of R and 0.1μg/kg of Cu. Ageing control mice received water twice daily by oral gavage.  **Results**  Using confocal microscopy and antibodies against DNA and histone we detected copious presence of extra-cellular cfChPs in brain of ageing mice. cfChPs were deactivated / eradicated following administration of R-Cu, and this was associated with down-regulation of several biological hallmarks of ageing in brain cells which included reduction in: 1) telomere attrition, 2) amyloid deposition, 3) DNA damage, 4) apoptosis, 5) inflammation, 6) senescence, 7) aneuploidy and 8) mitochondrial dysfunction. At a systemic level, R-Cu treatment led to significant reduction in blood levels of glucose, cholesterol and C-reactive protein.  **Conclusions**  cfChPs may act as global instigators of ageing and neurodegeneration, and therapeutic use of R-Cu may help to make healthy ageing an attainable goal. | **Abstract**  Page no 2 & 3 |
| **Introduction** | | | |
| **Background** |  | With progressively increasing longevity, the human race is facing a parallel increase in ageing related degenerative disorders which can severely compromise quality of life. It is predicted that, globally, the number of people age 60 years or above will grow by 38%, from 1 billion to 1.4 billion, outnumbering the youth during the next ten years [1]. The United Nations General Assembly has declared 2021–2030 the Decade of Healthy Ageing, with the ultimate goal to find therapeutic interventions which will simultaneously delay the many conditions associated with ageing [1, 2]. It is argued that healthy ageing should be considered as the ultimate preventive medicine [3]. Ageing is characterised by a myriad pathological processes which lead to gradual deterioration of structure and function of all cells and tissues of the body [4], and is associated with a multitude of degenerative disorders such as Alzheimer’s disease [5], cardiovascular diseases [6], diabetes [7], and cancer [8]. Although many theories of ageing have been advanced [9,10], none can comprehensively explain the numerous changes that accompany this multidimensional process.  DNA damage and chronic inflammation are two cardinal features of ageing [11, 12]. In this context, we have reported that cell-free chromatin particles (cfChPs) that are released from the billions of cells that die in the body every day, and enter into the extracellular compartments of the body, can be readily internalised by healthy cells wherein they inflict dsDNA breaks, activate apoptotic pathways and induce inflammatory cytokines [13, 14]. This has led us to hypothesise that repeated lifelong assault on healthy cells by cfChPs may be the underlying cause of ageing [15,16]. Our group has successfully isolated and characterised cfChPs from human serum, which upon EM examination revealed extensive size heterogeneity ranging between ~10nm and ~1000nm [13]. We have also reported that blood levels of cfChPs increase with age [17].  Our pre-clinical studies have led to the identification of a novel pro-oxidant combination of the nutraceuticals resveratrol (R) and copper (Cu) which deactivates cfChPs via the medium of oxygen radicals [18-20]. R is a well-known anti-oxidant which has been extensively researched for its health benefits [21]. However, and surprisingly, it acts as a pro-oxidant in presence of Cu, which is also a widely researched nutraceutical [22]. That oxygen radicals are generated when R and Cu are admixed was first demonstrated by Fukuhara *et al* [23]. They showed that R acts as a catalyst to reduce Cu (II) to Cu (I) resulting in generation of oxygen radicals which cleaved plasmid pBR322 DNA [24]**.** We have extended these findings to show that a combination of R and Cu can degrade genomic DNA and RNA [25], and can deactivate cfChPs *in vivo* by degrading their DNA component [18-20, 25]. We have further observed that, paradoxically, the DNA degrading activity of R-Cu increases as the molar concentration of Cu is gradually reduced with respect to R [25]. On the basis of this finding, in the present study, we kept the molar ratio of R : Cu at 1:10^-4^.  We have reported that a combination of R and Cu, when used at a ratio of 1:10^-4^, has therapeutic effects in several pre-clinical conditions associated with elevated extracellular levels of cfChPs [18-20]. For example, orally administered R-Cu can ameliorate toxic side effects of chemotherapy [18], and radiation therapy [19], and prevent bacterial endotoxin induced cytokine storm and fatality in mice [20]. Our early results also suggest that R-Cu is therapeutically effective in humans. An observational study showed that orally administered R-Cu to patients with severe Covid-19 led to reduction in mortality by nearly 50% [26]. We have also reported that grade III-IV mucositis could be significantly reduced by orally administered R-Cu in patients receiving high dose chemotherapy and bone marrow transplant for multiple myeloma [27]. R-Cu treatment also led to significant reduction in blood levels of inflammatory cytokines in that study.  Oxygen radicals that are generated upon oral administration of R-Cu are readily absorbed from the stomach to have systemic effects in the form of deactivation / eradication of extracellular cfChPs [18-20, 26, 27]. In the present study, we have taken advantage of cfChPs deactivating property of R-Cu to investigate whether prolonged administration of R-C to ageing mice will retard the hallmarks of ageing and neurodegeneration. The dose of R used in our study was 1mg/Kg, and that of Cu was 0.1μg/Kg, given by oral gavage twice daily. This dose of Cu was 20,000 times less, and that of R 5 times less, than those that have been used in pre-clinical studies to investigate their health promoting properties by other investigators [28, 29].  Using confocal microscopy and antibodies against DNA and histone we detected copious presence of extra-cellular cfChPs in brain of ageing mice, and observed that cfChPs were deactivated / eradicated following prolonged oral administration of R-Cu. Deactivation / eradication of cfChPs was associated with down-regulation of multiple biological hallmarks of ageing in brain cells. At a systemic level, R-Cu treatment led to significant reduction in blood levels of glucose, cholesterol and C-reactive protein. Taken together, our results suggest that cfChPs act as global instigators of ageing and neurodegeneration, and that therapeutic use of R-Cu may help to make healthy ageing an attainable goal. | **Background**  Paragraph 1 - 6  Page no. 4 - 6 |
| **Objectives** |  | To investigate whether cfChPs deactivating property of prolonged administration of R-Cu to ageing mice will retard the hallmarks of ageing and neurodegeneration. | **Background**  Paragraph 6  Page no. 6 |
|  | | | |
| **Ethical Statement** |  | The experimental protocol of this study was approved by the Institutional Animal Ethics Committee (IAEC) of Advanced Centre for Treatment, Research and Education in Cancer (ACTREC), Tata Memorial Centre, Navi Mumbai, India under permission No.16/2015. The experiments were carried out in compliance with the IAEC animal safety guidelines, and with those of ARRIVE mandates.  ACTREC- IAEC maintains respectful treatment, care and use of animals in scientific research. It aims that the use of animals in research contributes to the advancement of knowledge following the ethical and scientific necessity. All scientists and technicians involved in this study have undertaken training in ethical handling and management of animals under supervision of FELASA certified attending veterinarian. Animals were euthanised at appropriate time points under CO2 atmosphere by cervical dislocation under supervision of FELASA trained animal facility personnel. | **Methods**  Animal Ethics Approval  Paragraph 1& 2 |
| **Study Design** |  | The study comprised of 24 C57Bl/6 mice, 12 of which were male and 12 were female. Four mice of either sex were sacrificed when they were 3 months old, and acted as young controls. The remaining 16 mice (8 male and 8 female) were allowed to age until they were 10 months old and divided into two groups: 1) Ageing control mice (N=4 of each sex), and 2) R-Cu treated ageing mice (N=4 of each sex). Animals of both groups were sacrificed after 12 months when they were 22 months old.  R-Cu was administered twice daily by oral gavage for 12 months (from 10 months to 22 months) at a dose of 1mg/Kg of R and 0.1μg/kg of Cu. R, being insoluble in water, was administered as water suspension (100μL), and Cu was administered as a water-based solution (100μL). The ageing control mice were given water (100μL) twice daily by oral gavage. Our previous studies had shown this dose of R-Cu to be effective in multiple other pre-clinical conditions [18-20]. | **Methods**  Animals and R-Cu dosing  Paragraphs 2 & 3  Page no. 8 |
| **Experimental Procedures** |  | R-Cu was administered twice daily by oral gavage for 12 months (from 10 months to 22 months) at a dose of 1mg/Kg of R and 0.1μg/kg of Cu. R, being insoluble in water, was administered as water suspension (100μL), and Cu was administered as a water-based solution (100μL). The ageing control mice were given water (100μL) twice daily by oral gavage. Our previous studies had shown this dose of R-Cu to be effective in multiple other pre-clinical conditions [18-20].  Reduced physical activity and weight loss of mice were used as humane endpoints, and were scored twice every week. At appropriate time points mentioned above, blood was collected via retro-orbital route under isoflurane anaesthesia for serum separation. Animals were then euthanised under CO2 atmosphere by cervical dislocation under supervision of FELASA trained animal facility personnel. After euthanasia, brain was harvested from all animals, fixed in 10% formalin and paraffin blocks were prepared for further analysis. | **Methods**  Animal and R-Cu dosing  Paragraphs 3 & 4  Page no. 8 - 9 |
| **Experimental Animals** |  | Inbred C57Bl/6 mice obtained from the Institutional Animal Facility were maintained following by our Institutional Animal Ethics Committee standards. | **Methods**  Animal and R-Cu dosing  Paragraph 1  Page no. 8 |
| **Housing and Husbandry** |  | Inbred C57Bl/6 mice obtained from the Institutional Animal Facility were maintained following by our Institutional Animal Ethics Committee standards. They were housed in pathogen-free cages containing husk bedding under 12-h light/dark cycle with free access to water and food. The HVAC system was used to provide controlled room temperature, humidity and air pressure. | **Methods**  Animal and R-Cu dosing  Paragraph 1  Page no. 8 |
| **Sample Size** |  | The study comprised of 24 C57Bl/6 mice, 12 of which were male and 12 were female. Four mice of either sex were sacrificed when they were 3 months old, and acted as young controls. The remaining 16 mice (8 male and 8 female) were allowed to age until they were 10 months old and divided into two groups: 1) Ageing control mice (N=4 of each sex), and 2) R-Cu treated ageing mice (N=4 of each sex). Animals of both groups were sacrificed after 12 months when they were 22 months old. | **Methods**  Animal and R-Cu dosing  Paragraph 2  Page no. 8 |
| **Allocating animals to** **experimental groups** |  | The study comprised of 24 C57Bl/6 mice, 12 of which were male and 12 were female. Four mice of either sex were sacrificed when they were 3 months old, and acted as young controls. The remaining 16 mice (8 male and 8 female) were allowed to age until they were 10 months old and divided into two groups: 1) Ageing control mice (N=4 of each sex), and 2) R-Cu treated ageing mice (N=4 of each sex). Animals of both groups were sacrificed after 12 months when they were 22 months old.  At appropriate time points mentioned above, blood was collected via retro-orbital route under isoflurane anaesthesia for serum separation. Animals were then euthanised under CO2 atmosphere by cervical dislocation under supervision of FELASA trained animal facility personnel. After euthanasia, brain was harvested from all animals, fixed in 10% formalin and paraffin blocks were prepared for further analysis. | **Methods**  Animal and R-Cu dosing  Paragraph 2  Page no. 8 |
| **Experimental outcomes** |  | Experimental outcomes were analysed as follows:  1. Superoxide Dismutase (SOD) levels in brains cells by Immunofluorescence (IF) and SOD activity in serum by ELISA.  2. Detection of cfCh effusion into extra-cellular spaces of brain by fluorescence immune-staining and confocal microscopy.  3. Telomere abnormalities:  Telomere length estimation by qRT-PCR.  Telomere number and telomere aggregation by Q-FISH  4. β- amyloid deposition in brain by IF and BDNF in serum by ELISA  5. DNA damage, apoptosis and Inflammation in brain cells by IF  6. Senescence in brain cells by IF  7. Aneuploidy in brain cells IF  8. Mitochondrial dysfunction in brain cells IF  9. Systemic metabolic dysfunction by estimation of serum glucose, cholesterol (automated Bioanalyser) and CRP (ELISA) | **Methods**  Paragraphs 9 - 18  Page no. 9-12 |
| **Statistical Methods** |  | Statistical analyses were performed using GraphPad Prism 6 (GraphPad Software, Inc., USA. Version 6.0). Mean (± SEM) values for four mice in each group for both sexes were compared using non-parametric unpaired student’s t test, separately for both sexes. | **Methods**  Statistical analysis  Paragraph 19  Page no. 13 |
| **Results** | | | |
| **Baseline Data** |  | The results of aged mice and aged + R-Cu mice were compared with young control mice. | **Methods**  Statistical analysis  Paragraph 19  Page no. 13 |
| **Number Analyzed** |  | Mean (± SEM) values for four mice in each group for both sexes were compared separately. | **Methods**  Statistical analysis  Paragraph 19  Page no. 13 |
| **Outcomes and Estimations** |  | 1. R-Cu up-regulates SOD in brain cells and serum levels  2. R-Cu prevents effusion of cfCh particles into extra-cellular spaces of brain  3. R-Cu prevents telomere abnormalities in brain cells  4. R-Cu prevents telomere abnormalities in brain cells  5. R-Cu prevents amyloid deposition in brain and restores of BDNF levels in serum  6. R-Cu prevents DNA damage, apoptosis and inflammation in brain cells  7. R-Cu prevents senescence in brain cells  8. R-Cu prevents aneuploidy in brain cells  9. R-Cu prevents mitochondrial dysfunction in brain cells  10. R-Cu prevents systemic metabolic dysfunction | **Results**  Paragraph 1- 14  Page no. 13 - 18 |
| **Adverse Events** |  | Overall, we observed no adverse effects in mice which had been administered R-Cu for a period of one year. | **Discussion**  Paragraph 3  Page no. 21 |
| **Discussion** | | | |
| Interpretation / Scientific Implications |  | Our results suggest that cfChPs are global instigators of ageing and neurodegeneration, and that therapeutic use of R-Cu may help to make healthy ageing an attainable goal. | **Conclusion**  Paragraph 1  Page no. 22 |
| Generalizability / translation |  | We demonstrate for the first time that cfChPs derived from dying brain cells are abundantly present in the extracellular spaces of the ageing brain, and that they are virtually eliminated following prolonged treatment with R-Cu. The fact that elimination of cfChPs was associated with down-regulation of multiple biological hallmarks of ageing and neurodegeneration makes a strong case for a direct role of cfChPs in the aetiology of these pathological processes. We propose that cfChPs released from dying brain cells initiate a vicious cycle of more DNA damage, apoptosis and inflammation, setting in motion a low grade and unrelenting “cytokine storm” [65]. We propose that the latter, together with the other yet unknown harmful pleiotropic effects of cfChPs, are the underlying processes that define ageing. Our results suggest that these harmful effects can be prevented by deactivation / eradication of the offending cfChPs via the medium of oxygen radicals. We propose that oral administration of a combination of small quantities of R and Cu holds the promise of being an effective anti-ageing therapeutic combination. Whether R-Cu will be effective in retarding ageing and neurodegeneration in humans will have to await clinical trials in appropriate populations. It is to be noted, however, that our early results have shown that R-Cu is therapeutically effective in humans, albeit in context of other pathological situations [26, 27]. | **Discussion**  Paragraph 5  Page no. 21 & 22 |
| Funding |  | This study was supported by the Department of Atomic Energy, Government of India, through its grant CTCTMC to Tata Memorial Centre awarded to IM. The funding agency had no role in research design, collection, analysis, and interpretation of data and manuscript writing. | **Funding Section**  Paragraph 1  Page no. 24 |
